# Association of Hepatitis B Virus DNA Level and Follow-up Interval With Hepatocellular Carcinoma Recurrence

**DOI:** 10.1001/jamanetworkopen.2020.3707

**Published:** 2020-04-27

**Authors:** Wei Wang, Shilin-L. Tian, Hui Wang, Chun-Chun Shao, Yong-Zheng Wang, Yu-Liang Li

**Affiliations:** 1 Department of Interventional Medicine, The Second Hospital of Shandong University, Jinan, China; 2 Interventional Research Institute of Shandong University, Jinan, China; 3 Department of Interventional Medicine, Jilin Cancer hospital, Changchun, China; 4 Center of Evidence-based Medicine, Institute of Medical Sciences, The Second Hospital of Shandong University, Jian, China

## Abstract

**Importance:**

Antiviral treatment is important in hepatitis B virus (HBV)–related hepatocellular carcinoma (HCC) comprehensive therapy. A high HBV DNA level is an independent risk factor for HBV-related HCC, but no quantifiable clinical index is available to date.

**Objective:**

To evaluate the feasibility and availability of the novel HBV DNA quantitation-time index (HDQTI), which includes HBV DNA quantitation and follow-up, to predict HBV-related HCC prognosis.

**Design, Setting, and Participants:**

This retrospective prognostic study of patients with HCC from multiple centers in China was performed from January 1, 2002, to December 31, 2016. The median follow-up time was 18 months, and the longest follow-up time was 147 months. Data analysis was performed from January 1, 2017, to December 31, 2018.

**Main Outcomes and Measures:**

Clinical characteristics, antitumor management, antiviral treatment, HDQTI scores, follow-up information, and overall survival were recorded and analyzed. A receiver operating characteristic curve and accompanying area under the curve were calculated for HDQTI.

**Results:**

A total of 842 patients (mean [SD] age, 61.80 [9.85] years; 513 [60.9%] male) were included in the study. Of all included patients, 734 received no antiviral therapy before diagnosis (no previous diagnosis of HBV infection), 43 underwent nonstandard antiviral therapy, and 65 received regular antiviral therapy. Compared with the group without antiviral treatment, the Barcelona Clinic Liver Cancer (BCLC) stage was earlier (A:B:C, 73.8%:26.2%:0% to 5.7%:65.5%:28.8%, *P* < .001), the mean (SD) tumor size was smaller (2.89 [1.26] to 7.56 [3.28] cm, *P* < .001), the ratio of baseline HBV DNA level of more than 10^5^ copies/mL was lower (10.8% to 40.6%, *P* < .001), and the ratio of the α_1_-fetoprotein level more than 400 ng/mL was less (21.5% to 78.2%, *P* < .001) in the standard antiviral treatment group, whereas the nonstandard treatment group was between the 2 groups. Recurrence occurred in 39 of 109 BCLC stage A cases. Patients with HDQTI scores higher than 34 had high risk of recurrence; at this cutoff level, the sensitivity of the HDQTI was 76.9% and the specificity was 92.9%, with an area under curve of 0.928. Patients in various BCLC stages had similar trends in overall survival and HDQTI scores (BCLC stage A: HDQTI score <34, not applicable; HDQTI score ≥34, 44.0 months; 95% CI, 38.3-49.7 months; BCLC stage B: HDQTI score <34, 35.0 months; 95% CI, 33.3-36.7 months; HDQTI score ≥34, 17.0 months; 95% CI, 14.5-19.5 months; *P* = .002; BCLC stage C: HDQTI score <34, 18.0 months; 95% CI, 16.5-19.6 months; HDQTI scores ≥34, 10.0 months; 95% CI, 8.5-11.5 months; *P* = .005).

**Conclusions and Relevance:**

The findings suggest that the HDQTI can be used as an independent prognostic indicator of recurrence in HBV-related HCC. Shorter follow-up intervals and accurate imaging evaluation are recommended in patients with HDQTI scores of 34 or higher.

## Key Points

**Question:**

Is the novel hepatitis B virus DNA quantitation-time index a feasible and available means of assessing hepatitis B virus–related hepatocellular carcinoma prognosis?

**Findings:**

In this prognostic study of 842 patients with hepatocellular carcinoma, the hepatitis B virus DNA quantitation-time index, which combines hepatitis B virus DNA quantitation and follow-up, predicted disease recurrence with a 76.9% sensitivity and a 92.9% specificity.

**Meaning:**

The findings suggest that the hepatitis B virus DNA quantitation-time index can be used as an independent prognostic indicator of recurrence in hepatitis B virus–related hepatocellular carcinoma.

## Teaser

This prognostic study evaluates the feasibility and availability of a novel hepatitis B virus DNA index to assess the prognosis of patients with hepatitis B virus–related hepatocellular carcinoma.

## Introduction

Hepatitis B virus (HBV) is the most significant factor associated with the development of hepatocellular carcinoma (HCC), which currently constitutes the sixth most common cancer worldwide and the second most common cause of cancer-related death.^1^ Antiviral therapy studies^2,3^ have demonstrated the association of nucleoside or nucleotide analogues with delayed disease progression and with reduced incidence of HCC. Furthermore, in patients with developed HCC, concomitant antiviral therapy after initial tumor ablation is associated with improved survival and reduced tumor recurrence.^1^ Antiviral therapy is significantly associated with reduced HCC recurrence after R0 hepatic resection in patients with high or low preoperative HBV DNA levels.^4^ For patients with HBV-related HCC, compensated cirrhosis, and detectable HBV DNA, nucleoside or nucleotide analogue treatment after liver resection is cost-effective.^5^ Even for patients with HBV-related cirrhosis, oral antiviral drug treatment is recommended for reversing liver fibrosis and preventing HCC occurrence.^6^ HBV-related HCC also arises in noncirrhotic livers, supporting the notion that HBV plays a direct role in liver transformation by triggering common and virus-specific oncogenic pathways in addition to stimulating the host immune response and driving chronic liver necroinflammation.^7^ Better HBV follow-up and optimal HCC surveillance are associated with better overall survival.^8^ Even after curative HCC resection, tumor recurrence is high and is significantly associated with tumor size, vascular invasion, and intrahepatic metastasis.^9,10,11,12,13^ It is unclear which patients will have poor prognosis and require shorter follow-up intervals for earlier detection of recurrent lesions. Therefore, we designed a novel index to address these issues and assessed its effectiveness and feasibility in predicting HCC recurrence.

## Methods

### Patients

This retrospective prognostic study was approved by the Second Hospital of Shandong University Review Board and Ethical Committee. At the beginning of this retrospective analysis, the patients who could be contacted provided written informed consent; patients who could not provide informed consent were excluded from the study. The study followed the Standards for Reporting of Diagnostic Accuracy (STARD) reporting guideline.

The inclusion criteria of this study were presence of HCC confirmed by contrast-enhanced computed tomography (CT) or magnetic resonance imaging (MRI) and pathologic findings on α_1_-fetoprotein (AFP) testing or biopsy, HBV infection, regular follow-up and HBV DNA quantitation, Barcelona Clinic Liver Cancer (BCLC) stage A to C, and Child-Pugh scores A or B.^14^ Exclusion criteria were no HBV DNA quantitation record, other viral infections or mixed infections, BCLC stage D, Child-Pugh score C, HCC-related gastrointestinal bleeding before first treatment, HCC rupture, and other concurrent cancers. Patients with surgical indications were transferred to other departments and not included in this study

From January 1, 2002, to December 31, 2016, a total of 1043 patients with HCC were admitted to multiple interventional departments. Viral infection was detected in 967 patients, of whom 931 were infected with HBV, 25 with hepatitis C virus, and 11 with both. Of the 931 HBV-related HCC cases, 109 were BCLC stage A at first diagnosis, 524 were stage B, 276 were stage C, and 18 were stage D. Thirty-five patients were admitted to emergency departments for HCC rupture, and 17 had gastrointestinal bleeding history confirmed to be HCC related after admission. Thirteen patients could not provide HBV DNA quantitation results. Six patients were confirmed to have concurrent cancers, including prostate cancer, lung cancer, thymoma, breast carcinoma, gastric cancer, and bladder cancer. Therefore, there were 842 eligible patients.

### HCC Treatment

Comprehensive, individual interventional treatments were selected according to different conditions, including transcatheter arterial chemoembolization (TACE), radiofrequency ablation (RFA), microwave ablation (MWA), percutaneous ethanol injection (PEI), and brachytherapy, as well as some tumor-related complication treatments, such as percutaneous transhepatic cholangial drainage, duodenal stent implantation, and abdominal fluid drainage. For BCLC stage A, TACE followed by RFA or MWA was the most common treatment; for BCLC stage B, TACE-based comprehensive treatment; and for BCLC stage C, TACE combined with PEI or brachytherapy and sorafenib.

### Antiviral Therapy

Initial antiviral treatment included lamivudine, entecavir, adefovir dipivoxil, and tenofovir disoproxil fumarate. In case of drug resistance, another drug, such as adefovir, was added or substituted. The ultimate goal was to maximize long-term inhibition of HBV.

### HBV DNA Surveillance

HBV DNA was measured by reverse-transcription polymerase chain reaction (Quest Diagnostics), with a lower limit of detection of 500 copies/mL. Serial dilutions were performed for samples that exceeded 5.2 log_10_ copies/mL.

### HBV DNA Quantitation-Time Index 

The HBV DNA quantitation-time index (HDQTI) was designed to evaluate the influence of HBV according to the duration of its presence at high load during HCC treatment and was calculated as follows:
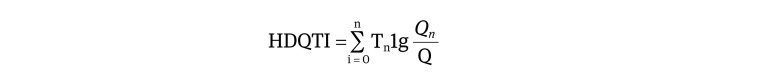
where *n* indicates number of follow-ups; *Q*, normal load of HBV DNA (500 copies/mL); *Q_n_*, HBV DNA load at nth follow-up; and *T_n_*, nth follow-up interval (months).

The HDQTI is the summation of the product of follow-up and the logarithm of the ratio of detected to normal HBV DNA load. When the HBV DNA load is normal, the ratio is 1 and its logarithm is 0, so the follow-up interval contributes nothing to the HDQTI. To reduce the effects of prolonged follow-up on the parameters, logarithmic conversion was applied. When the quantitative difference of HBV DNA between 2 reexaminations was too large (>4 orders of magnitude) while assessing parameters during follow-up, the original formula was modified. The root of the product of both values was obtained and divided by the normal value. In addition, for the review cycle of T0, patients with detailed data for regular follow-ups were assessed based on actual conditions. For newly diagnosed patients, T0 was selected for 3 months because the follow-up period was up to 3 months.

### Follow-up

All patients were followed up every 1 to 3 months. The median follow-up time was 18 months, and the longest follow-up was 147 months. During follow-up, HBV DNA load, hepatic function, whole blood cell count, AFP levels, and contrast-enhanced CT or MRI results were examined. Overall survival time (OS) and recurrence-free survival (RFS) were recorded.

### Statistical Analysis

Continuous variables were evaluated for normality by the Kolmogorov-Smirnov test. To assess differences between groups, the 2-tailed, unpaired *t* test, 1-way analysis of variance, or nonparametric testing was used for continuous variables, and the χ^2^ test or Fisher exact test was used for categorical variables. Differences were considered to be statistically significant at 2-sided *P* < .05. To predict stage A disease recurrence, the HDQTI score at the time of recurrence was used. For analyzing the association with prognosis, the HDQTI scores for all follow-up cycles were used. The Kaplan-Meier method was applied for survival analysis. A receiver operating characteristic (ROC) curve (recurrence status as categorical variable) was generated, and the accompanying area under the curve (AUC) was calculated for the HDQTI. Patients without disease recurrence during the follow-up were calculated as having no recurrence. The best cutoff value was determined using the Youden index. Data analysis was performed from January 1, 2017, to December 31, 2018. All statistical analyses were performed using SPSS, version 24.0 (IBM Inc).

## Results

### Study Population

A total of 842 patients (mean [SD] age, 61.80 [9.85] years; 513 [60.9%] male) were included in the study. All demographic and baseline clinical characteristics are presented in Table 1. Of these patients, 734 had never received antiviral therapy before diagnosis with HCC (no previous diagnosis of HBV infection), 43 received nonstandard antiviral therapy, and 65 received regular antiviral therapy. Significant differences were found among these 3 therapy groups with respect to median tumor size, BCLC stage, baseline HBV DNA levels, and AFP levels, whereas nonsignificant differences were found in Child-Pugh scores, albumin levels, and Impact-R platelet counts. The ratios of BCLC stages A:B:C were 5.7%:65.5%:28.8% for the no treatment group, 44.2%:48.8%:7.0% for nonstandard treatment group, and 73.8%:26.22%:0% for the standard treatment group (*P* < .001). Mean (SD) tumor size was 7.56 (3.28) cm for the no treatment group, 5.43 (2.53) cm in the nonstandard treatment group, and 2.89 (1.26) cm in the standard treatment group (*P* < .001). Hepatitis B virus DNA levels of more than 10^5^ copies/mL were found in 40.6% of the no treatment group, 20.9% of the nonstandard treatment group, and 10.8% of the standard treatment group and levels of 10^5^ copies/mL or less in 59.4% in the no treatment group, 79.1% in the nonstandard treatment group, and 89.2% in the standard treatment group. AFP levels greater than 400 ng/mL (to convert to micrograms per milliliter, multiply by 1) were found in 78.2% of the no treatment group, 23.3% of the nonstandard treatment group, and 21.5% of the standard treatment group and of 400 ng/mL or less in 21.8% of the no treatment group, 76.7% of the nonstandard treatment group, and 78.5% of the standard treatment group (*P* < .001). Child-Pugh stage A was found in 74.4% in the no treatment group, 74.4% of the nonstandard treatment group, and 72.3% of the standard treatment group and B in 25.6% of the no treatment group, 25.6% of the nonstandard treatment group, and 27.7% of the standard treatment group. The mean (SD) bilirubin level was 14.60 (4.80) g/L (to convert to micromoles per liter, multiply by 17.104) in the no treatment group, 13.84 (4.15) in the nonstandard treatment group, and 14.11 (3.30) in the standard treatment group (Table 2).

**Table 1.** Demographic and the Baseline Clinical Characteristics

Characteristic	No. (%) of patients (N = 842)
Age, y	
≤60	391 (46.4)
>60	451 (53.6)
Sex	
Male	513 (60.9)
Female	329 (39.1)
Tumor size, cm	
≤5	380 (45.1)
>5	462 (54.9)
Multinodular	
Single	134 (15.9)
Multiple	708 (84.1)
BCLC stage	
A	109 (12.9)
B	519 (61.7)
C	214 (25.4)
Serum AFP, ng/mL	
≤400	244 (29.0)
>400	598 (71.0)
HDQTI score	
34	570 (67.7)
≥34	272 (32.3)

Abbreviations: AFP, α_1_-fetoprotein; BCLC, Barcelona Clinic Liver Cancer; HDQTI, hepatitis B virus DNA quantitation-time index.

SI conversion factors: To convert AFP to micrograms per liter, multiply by 1.

**Table 2.** Association of Antiviral Treatment History and Basic Clinical Characteristics^a^

Characteristic	Antiviral treatment			*F* or χ^2^ test^b^	*P* value
	None (n = 734)	Nonstandard (n = 43)	Standard (n = 65)		
BCLC stage					
A	42 (5.7)	19 (44.2)	48 (73.8)	289.93	<.001^c^^,^^d^^,^^e^
B	481 (65.5)	21 (48.8)	17 (26.2)		
C	211 (28.8)	3 (7.0)	0		
Tumor size, mean (SD), cm	7.56 (3.28)	5.43 (2.53)	2.89 (1.26)	72.91	<.001^d^^,^^e^
Baseline HBV DNA level					
>10^5^ copies/mL	298 (40.6)	9 (20.9)	7 (10.8)	27.91	<.001^c^^,^^d^
≤10^5^ copies/mL	436 (59.4)	34 (79.1)	58 (89.2)		
Child-Pugh stage					
A	546 (74.4)	32 (74.4)	47 (72.3)	0.136	.93
B	188 (25.6)	11 (25.6)	18 (27.7)		
AFP					
>400 ng/mL	574 (78.2)	10 (23.3)	14 (21.5)	143.39	<.001^c^^,^^d^
≤400 ng/mL	160 (21.8)	33 (76.7)	51 (78.5)		
Bilirubin, mean (SD), g/L	14.60 (4.80)	13.84 (4.15)	14.11 (3.30)	0.82	.44
Platelets, mean (SD), ×10^3^/μL	136.63 (29.78)	129.37 (22.11)	133.52 (31.74)	1.47	.23

Abbreviations: AFP, α_1_-fetoprotein; BCLC, Barcelona Clinic Liver Cancer; HBV, hepatitis B virus.

SI conversion factors: To convert AFP to micrograms per liter, multiply by 1; bilirubin to micromoles per liter, multiply by 17.104; platelets to ×10^9^/L, multiply by 1.

^a^ Data are presented as number (percentage) of patients unless otherwise indicated.

^b^ *F* for tumor size, bilirubin level, and platelet count and χ^2^ for BCLC stages and baseline HBV DNA level.

^c^ Difference was significant between the no antiviral treatment group and the nonstandard antiviral treatment group.

^d^ Difference was significant between the no antiviral treatment group and the standard antiviral treatment group.

^e^ Difference was significant between the nonstandard antiviral treatment group and the standard antiviral treatment group.

### Antiviral Therapy

Initial antiviral treatment included lamivudine, entecavir, adefovir, and tenofovir. With anti-HBV therapy, HBV DNA became undetectable in most patients at a median of 8 months (range, 3-12 months). A given drug was administered to those with low baseline HBV DNA levels, and as long as levels remained undetectable, the medication was not changed. All patients who maintained undetectable HBV DNA levels throughout antiviral therapy were followed up at 3-month intervals. If HBV DNA levels became detectable, drug resistance was considered, and HBV genotyping and phenotyping were required. Thirteen cases exhibited viral breakthrough and drug resistance. When drug resistance occurred, another drug was added or substituted; these selections are listed in Table 3. The ultimate goal of treatment was to maximize long-term inhibition of HBV.

**Table 3.** Antiviral Treatment Resistance and Alternative Therapies

Drug	Alternative treatment	No. of patients
Lamivudine	Switch to tenofovir	2
	Add adefovir when tenofovir is unavailable	0
Adefovir dipivoxil	Switch to tenofovir if available and add a second drug	6
	rtN236T substitution is present, add entecavir	1
	Without lamivudine resistance detected by sensitive assays for one with prior lamivudine exposure, switch to entecavir	1
	rtA181V/T substitution is present, alone or in combination with rtN236T, switch to tenofovir plus entecavir	0
Telbivudine	Add or switch to tenofovir	1
Entecavir	Add tenofovir	2
Tenofovir disoproxil fumarate	Hepatitis B virus genotype and phenotyping is required, entecavir may be added	0

### Anti-HCC Treatment 

In the present study, HBV DNA levels in 23 patients were significantly higher after anti-HCC treatment than before. Among these patients, 17 received TACE, 4 had PEI, 1 received RFA, and 1 underwent brachytherapy. Twenty patients (16.9%) received nonstandard or no antiviral treatment, and only 3 patients (0.4%) who received standard antiviral treatment experienced HBV reactivation. Furthermore, 721 of the 724 patients (99.6%) who received standard antiviral treatment and 98 of 118 (83.1%) who received nonstandard or no treatment did not experience HBV reactivation (χ^2^ = 98.27; *P* < .001), indicating that antiviral treatment concurrent with HCC treatment was associated with a reduced risk of HBV reactivation.

### HDQTI as an Independent Prognostic Indicator of HCC Recurrence

In patients with BCLC stage A disease, TACE followed by RFA or MWA was the most common treatment regimen. Enhanced MRI and diffusion or positron emission tomography–CT were used at follow-up 1 month after treatment to ensure that the lesions were inactive. A total of 109 patients receiving this treatment regimen had complete response. Follow-up intervals were between 1 and 3 months depending on whether HBV DNA levels were detectable. Hepatocellular carcinoma recurred in 39 patients, including all patients with drug-resistant HBV infection. TACE was selected as the first choice for rescue therapy. Additional RFA or WMA was also selected when the recurrent lesion was smaller than 3 cm. The HDQTI was recorded in all cured patients; the ROC curve of the HDQTI and recurrence is shown in the Figure, A, with an AUC of 0.928 (95% CI, 0. 91-0.99; *P* < .001; sensitivity, 0.769; specificity, 0.929). Disease tended to recur in patients with HDQTI scores higher than 34 (χ^2^ = 101.15, *P* = .003). When the HDQTI score was 34, sensitivity was 76.9% and specificity was 92.9%.

**Figure.**
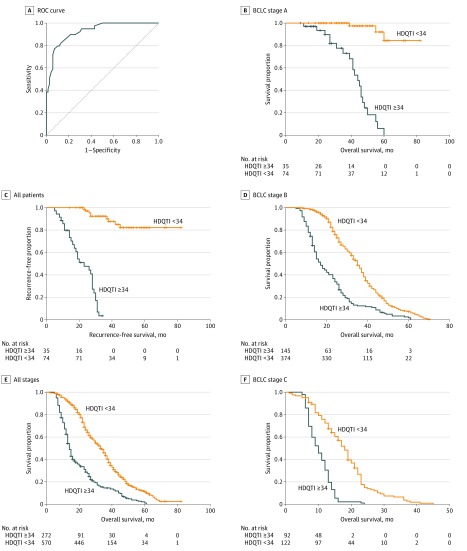
Hepatitis B Virus (HBV) DNA Quantitation-Time Index (HDQTI) for HBV-Related Hepatocellular Carcinoma (HCC) Therapy A, Receiver operating characteristic (ROC) curve of the HDQTI score in estimating cured HBV-related HCC recurrence in patients with Barcelona Clinic Liver Cancer (BCLC) stage A disease. The area under curve was 0.928 (95% CI, 0.91–0.99) The diagonal line indicates the fase-positive rate. B, Kaplan-Meier curves of the HDQTI in patients with BCLC stage A HBV-related HCC recurrence-free survival. C-F, Kaplan-Meier curves of the HDQTI in HBV-related HCC overall survival for all patients (C), patients with BCLC stage A disease (D), patients with BCLC stage B disease (E), and patients with BCLC stage C disease (F). Hash marks indicate censored data.

### Association of the HDQTI With HCC RFS and OS

HCC recurred in 39 of the 109 cured patients with BCLC stage A disease. The median RFS rate in patients with BCLC stage A disease with HDQTI scores less than 34 was not available because of recurrence in less than half the cases, but recurrence rates were high in patients with HDQTI scores greater than 34 (23.0 months; 95% CI, 14.5–31.5 months) (Figure, B). Overall survival rates were significantly different between patients with HDQTI scores lower (33.0 months; 95% CI, 30.7-35.3 months) and higher (14.0 months; 95% CI, 13.1-14.9 months; Figure, C) than 34 (*P* = .002). Patients with disease of various BCLC stages had similar OS rates and HDQTI score (BCLC stage A: HDQTI score <34, not applicable; HDQTI score ≥34, 44.0 months; 95% CI, 38.3-49.7 months; BCLC stage B: HDQTI score <34, 35.0 months; 95% CI, 33.3-36.7 months; HDQTI score ≥34, 17.0 months; 95% CI, 14.5-19.5 months; *P* = .002; BCLC C: HDQTI score <34, 18.0 months; 95% CI, 16.5-19.6 months; HDQTI score ≥34, 10.0 months; 95% CI, 8.5-11.5 months; *P* = .005) (Figure, D-F).

## Discussion

In China, HBV is the most common carcinogenic risk factor of HCC.^15^ Approximately 10% of patients infected with HBV develop liver cirrhosis, and a portion of these patients ultimately develop HCC. This finding represents disease progression significantly different from that in the West, where HCC is most often caused by alcoholic cirrhosis. Alcohol represents the most common cause of HCC in Italy and the US, accounting for 32% of all HCC cases in Italy and 35% in the US.^10^ High HBV DNA level is the main risk factor for hepatocarcinogenesis.^16^ Hepatitis B virus precure and core region mutations might be associated with HCC tumorigenesis.^17^ In this study, when patients received a diagnosis, the BCLC stage was associated with antiviral treatment and baseline HBV DNA level. Patients who received regular antiviral treatment received a diagnosis earlier with smaller tumors likely because of more frequent follow-up. Hepatic function, AFP levels, and platelet counts were not significantly different among the antiviral treatment groups.

The current, potent nucleoside or nucleotide analogues are associated with reduced but not eliminated HCC risk in HBV-infected patients probably because of risk factors not amenable to change by antiviral therapy or to events before treatment initiation.^18^ In the present study, control of the HBV DNA level and duration were significantly associated with HCC recurrence and OS. Although the of association high HBV DNA level with recurrence and OS was strong, the association was weaker after timely drug adjustment. Duration of high HBV DNA load was also important. To evaluate the association of HBV DNA load and duration with HCC recurrence and OS, we designed the novel HDQTI, which combines HBV DNA quantitation and follow-up. To eliminate instrumental error and for statistical convenience, we chose the ratio of HBV DNA load detected at follow-up to normal load and multiplied the logarithm of this ratio by the follow-up interval (months). The HDQTI is the summation of this calculation for each follow-up interval. According to this equation, when the detected HBV DNA level is normal, its contribution to the HDQTI in the follow-up interval is 0. The higher the virus quantity, the longer the follow-up duration and the higher the HDQTI score.

The HDQTI scores were significantly associated with recurrence of HCC and RFS. The ROC curve for the HDQTI had an AUC of 0.928. When the HDQTI score was higher than 34, the risk of recurrence was higher than when the HDQTI score was less than 34, with a sensitivity of 0.769 and a specificity of 0.929. Thus, patients with HDQTI scores less than 34 had a lower risk of recurrence. These findings suggest that HDQTI scores of 34 or higher warrant shorter follow-up intervals, and contrast MRI or positron emission tomography–CT instead of CT should be used to reduce chances of missed diagnoses. In addition, the HDQTI scores were associated with OS in all patients with various BCLC stages. These results suggest that the HDQTI can also be used as an independent prognostic indicator. Some patients had low HDQTI scores but short survival times mostly because of very high HBV DNA levels during short follow-up durations. This finding suggests that uncontrolled HBV infection hampers the therapeutic effects of antitumor treatment. Concurrent antiviral treatment may be associated with a reduced risk of HCC recurrence.^4^

The present study found that several antitumor treatments, including TACE, RFA, MWA, PEI, and brachytherapy, were associated with HBV reactivation, which is associated with recurrence of HCC and OS, consistent with previous reports.^19,20,21,22,23^ Combining antiviral treatment with conventional antitumor therapy is significantly associated with a reduced HBV reactivation.^24,25^ When treating liver cancer (eg, by TACE), some patients developed hepatitis B flares, and their risk was reduced by the use of antiviral drugs. If antiviral drugs were not applied previously, timely medication review and adjustment were performed, with a return to normal after treatment. If antiviral treatment was received, drug resistance tests were performed immediately and the medication regimen was adjusted according to findings to increase liver protection by supportive treatment. The HBV DNA level represents an important variable in evaluating HBV reactivation^26^ and is nonlinearly, dose-dependently associated with HCC.^27^ The interval between the peak serum HBV DNA load and hepatitis onset was variable. Nucleoside or nucleotide analogue–induced hepatitis B e antigen (HBeAg) seroconversion is associated with higher risk of HBV reactivation and HBeAg seroreversion than is spontaneous HBeAg seroconversion.^28^ Antiviral drug resistance might be another factor associated with HBV reactivation. Regular HBV DNA surveillance could reveal changes in HBV DNA levels, and timely adjustment of the antiviral treatment may be associated with reduced variations. Variation is the most common cause of high HBV DNA loads. The F30V variation is tightly correlated with HBV-induced HCC in vivo. Although F30V hampers HBV replication, the variation may be associated with hepatocyte survival, potentially allowing persistent production of viral progeny and initiating HBV-driven hepatocarcinogenesis.^29^ T-cell dysfunction is associated with chronic HBV infection and HCC, possibly through the mechanism of chronic HBV infection–specific deregulated genes regulating T-cell function.^30^ Hepatitis B virus reactivation, irregular antiviral treatment, or overdue drug adjustment after resistance development are associated with OS. Possible mechanisms of the benefits of antiviral treatment in HCC management include reducing the risk of HBV reactivation and liver function damage, decreasing mortality associated with hepatic failure, ameliorating HBV-induced hepatitis, and promoting the recovery of rescued liver tissue, which provide more chances for patients to receive local and systemic treatment during recurrence, prolonging OS.^31,32^ In patients with high levels of active HBV replication, antiviral treatment may be associated with elimination of the virus after antitumor management and with improved OS. In patients with low HBV DNA levels, antiviral treatment is controversial.^31^ In the present study, we considered antiviral treatment necessary because it was significantly associated with reduced HCC recurrence and prolonged OS.

The objectives of antiviral treatment are to minimize HBV infection and reduce hepatocyte inflammation, necrosis, and hepatic fibrosis. Irrespective of whether HBV DNA levels are normal or high, we believe that all patients with HBV-related HCC should receive antiviral treatment. Regular HBV DNA surveillance and antiviral treatment are recommended from diagnosis. HBV variation tests and antiviral treatment adjustments should be considered in case of antiviral drug resistance. When the HDQTI score is 34 or higher, cured HCC tended to recur with poor prognosis. Shortening the follow-up interval and accurate imaging evaluation should be considered.

Several factors are considered to be prognostic indicators of HBV-related HCC. Postoperative circulating tumor cell counts (>2/mL and >5/mL) and changes in circulating tumor cell counts may be independent prognostic indicators of progression-free survival in patients with HBV-related HCC, with the reduction in the number of circulating tumor cells showing better predictive performance.^33^ A clinical trial^34^ found that elevated heme-oxygenase 1 expression is associated with favorable disease-free survival in patients with HBV HCC who have undergone hepatectomy.

### Limitations

This study has limitations. The lack of comprehensive analysis of HDQTI, AFP, circulating tumor cells,^29^ and imaging characteristics to estimate HCC recurrence is a limitation of this study. In addition, the total data set should ideally have been divided into training and validation data sets to identify the best cutoff for the HDQTI and to assess the performance of the selected cutoff, respectively, which was not done in the current study. Furthermore, a retrospective design was adopted. Patients receiving antiviral therapy were regularly followed up, which promoted the early detection of tumors. Therefore, additional prospective, multicenter randomized clinical trials with multifactor analysis are warranted to confirm the current results.

## Conclusions

The findings suggest that HDQTI can be used as an independent prognostic indicator of HCC recurrence, RFS, and OS in HBV-related HCC. Shortening follow-up intervals and accurate imaging evaluation are recommended for patients with HDQTI scores of 34 or higher.
